# Deployment of Real-time Natural Language Processing and Deep Learning Clinical Decision Support in the Electronic Health Record: Pipeline Implementation for an Opioid Misuse Screener in Hospitalized Adults

**DOI:** 10.2196/44977

**Published:** 2023-04-20

**Authors:** Majid Afshar, Sabrina Adelaine, Felice Resnik, Marlon P Mundt, John Long, Margaret Leaf, Theodore Ampian, Graham J Wills, Benjamin Schnapp, Michael Chao, Randy Brown, Cara Joyce, Brihat Sharma, Dmitriy Dligach, Elizabeth S Burnside, Jane Mahoney, Matthew M Churpek, Brian W Patterson, Frank Liao

**Affiliations:** 1 University of Wisconsin - Madison Madison, WI United States; 2 Loyola University Chicago Chicago, IL United States

**Keywords:** clinical decision support, natural language processing, medical informatics, opioid related disorder, opioid use, electronic health record, clinical note, cloud service, artificial intelligence, AI

## Abstract

**Background:**

The clinical narrative in electronic health records (EHRs) carries valuable information for predictive analytics; however, its free-text form is difficult to mine and analyze for clinical decision support (CDS). Large-scale clinical natural language processing (NLP) pipelines have focused on data warehouse applications for retrospective research efforts. There remains a paucity of evidence for implementing NLP pipelines at the bedside for health care delivery.

**Objective:**

We aimed to detail a hospital-wide, operational pipeline to implement a real-time NLP-driven CDS tool and describe a protocol for an implementation framework with a user-centered design of the CDS tool.

**Methods:**

The pipeline integrated a previously trained open-source convolutional neural network model for screening opioid misuse that leveraged EHR notes mapped to standardized medical vocabularies in the Unified Medical Language System. A sample of 100 adult encounters were reviewed by a physician informaticist for silent testing of the deep learning algorithm before deployment. An end user interview survey was developed to examine the user acceptability of a best practice alert (BPA) to provide the screening results with recommendations. The planned implementation also included a human-centered design with user feedback on the BPA, an implementation framework with cost-effectiveness, and a noninferiority patient outcome analysis plan.

**Results:**

The pipeline was a reproducible workflow with a shared pseudocode for a cloud service to ingest, process, and store clinical notes as Health Level 7 messages from a major EHR vendor in an elastic cloud computing environment. Feature engineering of the notes used an open-source NLP engine, and the features were fed into the deep learning algorithm, with the results returned as a BPA in the EHR. On-site silent testing of the deep learning algorithm demonstrated a sensitivity of 93% (95% CI 66%-99%) and specificity of 92% (95% CI 84%-96%), similar to published validation studies. Before deployment, approvals were received across hospital committees for inpatient operations. Five interviews were conducted; they informed the development of an educational flyer and further modified the BPA to exclude certain patients and allow the refusal of recommendations. The longest delay in pipeline development was because of cybersecurity approvals, especially because of the exchange of protected health information between the Microsoft (Microsoft Corp) and Epic (Epic Systems Corp) cloud vendors. In silent testing, the resultant pipeline provided a BPA to the bedside within minutes of a provider entering a note in the EHR.

**Conclusions:**

The components of the real-time NLP pipeline were detailed with open-source tools and pseudocode for other health systems to benchmark. The deployment of medical artificial intelligence systems in routine clinical care presents an important yet unfulfilled opportunity, and our protocol aimed to close the gap in the implementation of artificial intelligence–driven CDS.

**Trial Registration:**

ClinicalTrials.gov NCT05745480; https://www.clinicaltrials.gov/ct2/show/NCT05745480

## Introduction

### Background

As of 2017, >95% of the hospitals in the United States adopted an electronic health record (EHR), and >80% are collecting electronic clinical notes [1]. Clinical decision support (CDS) and intelligent data-driven alerts are part of federal incentive programs for Meaningful Use [2,3]. With the increasing capacity of EHR data and financial incentives to improve quality care, hospitals are increasingly well equipped to leverage computational resources to improve case identification and care throughput [4].

The unstructured narrative of EHRs provides a rich source of information on patients’ conditions that may serve as CDS tools. Detailed medical information is routinely recorded in providers’ intake notes. However, this information is neither organized nor prioritized during routine care for augmented intelligence at the bedside. Moreover, clinical notes’ free-text format hinders efforts to perform analytics and leverage the large domain of data. The computational methods of natural language processing (NLP) can derive meaning from clinical notes, from which machine learning algorithms can screen for conditions such as opioid misuse.

In 2020, overdose deaths from opioid misuse soared to an all-time high, with a record 93,000 deaths nationwide during the pandemic year [5]. Substance misuse ranks second among the principal diagnoses for unplanned 7-day hospital readmission rates [6]. Screening for patients at risk for opioid use disorders is not part of the admission routine at many hospitals, and many hospitalized patients in need are never offered opioid treatment. The high prevalence rate of substance use disorders in hospitalized adults exceeds the rates in the general population or outpatient setting and reveals the magnitude of this lost opportunity [7]. We previously trained a convolutional neural network (CNN) that outperformed a rule-based approach and other machine learning methods for screening opioid misuse in hospitalized patients. The CNN substance misuse classifier had >80% sensitivity and specificity and demonstrated that clinical notes captured during hospitalization may be used to screen for opioid misuse [8].

There remains a paucity of evidence on the implementation of clinical NLP models in an interoperable and standardized CDS system for health operations and patient care [9]. The interactions among an artificial intelligence (AI) system, its users, its implementation, and the environment influence the AI intervention’s overall potential effectiveness. Few health systems have been able to accommodate the complexities of an NLP deep learning model integrated into an existing operational ecosystem and EHR [10]. Much of the literature on NLP-driven CDS has described retrospective studies [11,12] outside the clinical workflow or simulated clinical environments [13,14]. Others have used NLP for information extraction efforts aimed at quality improvement without direct integration into the clinical workflow and operations [15,16]. Few provide a real-time NLP system but do not share an implementation framework or pipeline details to ensure fidelity and reproducibility [17]. Although the field of AI-driven CDS is growing, sharing knowledge in development and operations for health care delivery is lacking in best practices for processes and technologies in application planning, development, delivery, and operations.

### This Study

This protocol describes a cloud service designed to ingest, process, and store clinical notes as standardized and interoperable messages from a major EHR vendor in an elastic cloud computing environment. We subsequently demonstrate the use of multiple open-source tools, including an open-source NLP engine for processing EHR notes and feeding them into a deep learning algorithm for screening for opioid misuse. Our resultant NLP and deep learning pipeline can process clinical notes and provide decision support to the bedside within minutes of a provider entering a note into the EHR.

To our knowledge, this is the first protocol for a bedside implementation of an NLP-driven CDS tool. We expect that our protocol will serve as a guide for other health systems to leverage open-source tools across interoperable data standards and ontologies. We provide an implementation framework and a cost-effectiveness analysis of a tool developed for the automated screening of hospitalized adults for opioid misuse. We aimed to describe a hospital-wide protocol and computing architecture for implementing a real-time NLP-driven CDS tool.

## Methods

### Hospital Setting and Study Period

The NLP CDS tool was implemented at the University of Wisconsin (UW) Hospital across the surgical and medical hospital inpatient wards. The EHR system used at the UW Health is Epic (Epic Systems Corp). The tool was designed for hospitalized adults (aged ≥18 years) and was assessed using a pre-post quasi-experimental study design over 30 months (24 months of usual care and 6 months for the implementation of automated screening). The study was a quality improvement initiative by the health system to provide an automated hospital-wide screening system for opioid misuse and was registered on ClinicalTrials.gov (NCT05745480).

### Preintervention Period: Usual Care With Ad Hoc Addiction Consultations

The UW Hospital launched an Addiction Medicine inpatient consult service in 1991 to address the high prevalence of substance use disorders among hospitalized adults. A screening, brief intervention, and referral to treatment program [18] was instituted for alcohol misuse. Screening, intervention flow sheets, and consult order sets were built into EHR-driven workflows for inpatient nurses and social workers for alcohol screening using the Alcohol Use Disorders Identification Test–Concise [19], a best practice alert (BPA) for patients at risk of alcohol use disorder, and order sets for withdrawal treatment. For other drugs, a single screening item queries “marijuana or other recreational drug use,” but no formal screening process was in place specifically targeting opioid misuse. For patients at risk of an opioid use disorder, the practice was ad hoc consultations at the discretion of the primary provider.

### Postintervention Period: Computing Architecture and Real-time Implementation

#### Overview

**Figure 1:**
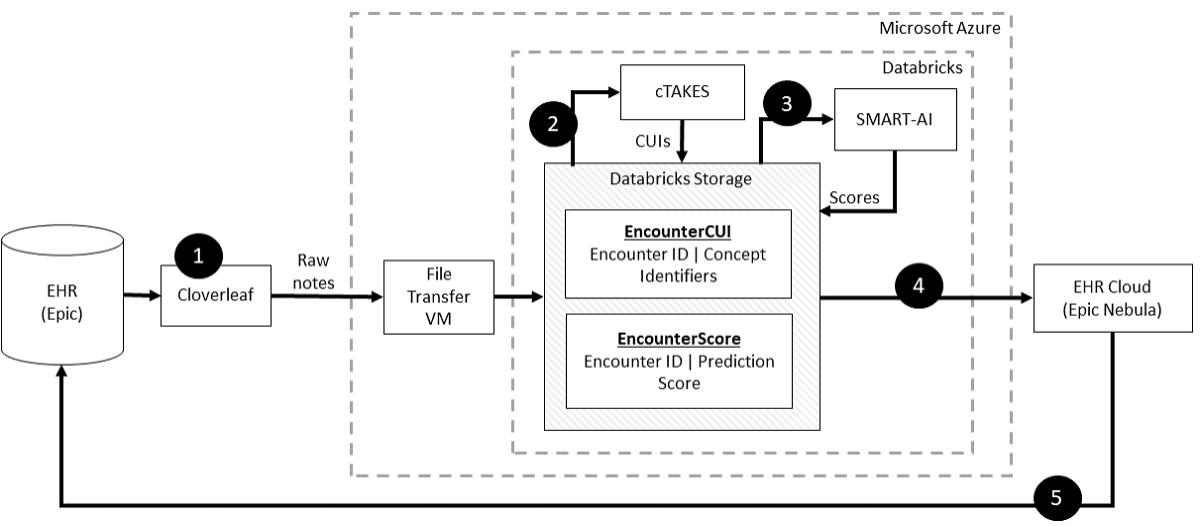
Step-by-step implementation of clinical natural language processing (NLP) pipeline. Step 1 ran a scheduled program to ingest notes from the EHR for each patient organized the notes, and relayed them via an HL7 data feed (Cloverleaf) into the cloud computing environment and data lake (Microsoft Azure and Databricks) onto a VM (Step 2). The NLP engine (cTAKES) processed the text stored on the VM and mapped them to medical concepts from the National Library of Medicine’s metathesaurus (CUIs). The machine learning model received the CUIs as inputs and stored the results in DataBricks. At regular intervals, a custom Python script in Databricks performed the text extraction and linguistic feature engineering via cTAKES and stored CUIs with the appended data of patient identifiers. The CUIs served as the input to the machine learning model (SMART-AI) at the encounter level. The output of prediction probabilities and classification was stored in a Databricks table (Step 3). An API call from the EHR cloud is made to determine whether the cutpoint threshold from the machine learning model was met to trigger a BPA. In Step 4, the EHR cloud made an HTTP call to Databricks to request the score. The score was returned to the EHR cloud and subsequently delivered as a BPA when the provider opened the patient’s chart in our on-premise instance of the EHR (Step 5). API: application programming interface; BPA: best practice alert; cTAKES: Clinical Text Analysis and Knowledge Extraction System; CUI: concept unique identifier; EHR electronic health record; HL7: Health Level 7; SMART-AI: Substance Misuse Algorithm for Referral to Treatment Using Artificial Intelligence; VM: virtual machine.

The technical architecture that enabled the real-time NLP CDS tool incorporated industry-leading and emerging technological capabilities. Figure 1 details the overall NLP CDS infrastructure that exported the notes from the EHR, organized them, and fed them into an NLP pipeline; input the processed text features into the opioid screener deep learning model; and delivered the resultant scores back to the bedside EHR as a BPA. The final architecture was a real-time NLP CDS tool, and the 6 components of the architecture are further detailed in the subsequent sections.

#### Component 1: Transferring Clinical Notes From the EHR to Cloud Computing

Health Level 7 (HL7) refers to the standards for transferring health care data between data sources. Cloverleaf (Infor Cloverleaf Integration Suite) was the UW’s vendor solution that served as an application programming interface (API) gateway for accessing the clinical narratives in the EHR using HL7 version 2. To initialize the data feed, a UW Health interface analyst created a new entry in the Cloverleaf vendor software detailing the desired record information, which included clinical note text and identifiers. The analyst then “activated” the data feed, which began a continuous Transmission Control Protocol message–generating process. The Transmission Control Protocol messages were communicated using the HL7 application protocol to the Azure Virtual Machine (version 2022; Microsoft Corp) host at a port designated by the data science engineering team. This port was reserved by a NET program (the “TCP listener”), which wrote the message to the cloud file system and replied to the Cloverleaf server with acknowledgment messages, conforming to the HL7 version 2 specification. Ultimately, the API extracted clinical notes from Epic and transferred them to a Microsoft Azure cloud computing environment that was under a business associate agreement with the UW. On-premise relays with the File Transfer Protocol were used to transfer the clinical notes to a specified location in the Azure cloud environment.

#### Component 2: Cloud Analytic Computing Platform

In the Microsoft Azure framework [8], the UW Health invoked the Databricks (Databricks Inc) analytic resources and services for scalable computing, data storage, and querying. The open-source tools from the NLP engine and our trained, publicly available machine learning model were hosted in Databricks. The machine learning model life cycle management (MLFlow) tool in Databricks supported the data flow for the deep learning model. MLFlow created and scored models when clinical notes were received and subsequently reported the results upon request. The final infrastructure was a scalable and failure-resistant environment for analytic computations.

#### Component 3: NLP Pipeline

The Clinical Text Analysis and Knowledge Extraction System (cTAKES; Apache Software Foundation) was built on multiple open-source Apache projects and incorporated technologies with the Unstructured Information Management Architecture framework and the Apache OpenNLP NLP toolkit [20]. This configuration contained several engines for sentence detection, tokenization, part-of-speech tagging, concept detection, and normalization to extract information from the clinical narrative in the EHR. We did not use the negation module because it was not used in the current use case; however, this can be turned on for other use cases. cTAKES is one of the most ubiquitous NLP engines used in the clinical domain [21]. cTAKES provided named entities from the free text that were mapped from the National Library of Medicine’s Unified Medical Language System (UMLS), which is a repository of groups of words with relevant clinical contexts (eg, drugs, diseases, symptoms, anatomical sites, and procedures). Each named entity was mapped to a concept unique identifier (CUI) using the UMLS Systemized Nomenclature of Medicine–Clinical Terms and medical prescription normalized ontologies. For instance, “heroin misuse” from the text was assigned C0600241 as its CUI, which was different from the CUI assigned to “history of heroin misuse,” C3266350. For generalizability, we used the default cTAKES pipeline [22].

As clinical notes were entered into the EHR for an individual patient, Cloverleaf relayed the notes via HL7 from the Epic EHR and used the Azure File Transfer Protocol server running on a virtual machine to place them at a known location within the Azure cloud environment. In 15-minute intervals, Databricks triggered a custom Python script to extract the text and fed it into the cTAKES pipeline to map and extract the CUIs. The CUIs were stored in the Azure Data Lake with appended data, including patient ID, encounter ID, and note time stamp, and were ready to be fed into any machine learning model. The code executed for the pipeline consisted of several services that operated independently and communicated through data stores. These services were “always on,” but each had a trigger condition that initiated the code execution. The pseudocode for these services is provided in Multimedia Appendix 1.

#### Component 4: Text Feed From the NLP Pipeline Into the Deep Learning Model

We previously published a substance misuse screening algorithm using CUIs fed into a CNN called the Substance Misuse Algorithm for Referral to Treatment Using Artificial Intelligence (SMART-AI) [8]. SMART-AI was trained on the first 24 hours of all clinical notes entered into the EHR, starting from the patient’s arrival time. This approach provided sufficient time not only for robust training but also for the addiction consult service to intervene before hospital discharge. For ease of implementation, the model was not trained on any specific note type and followed a time stamp approach for all notes filed within 24 hours of arrival at the hospital. SMART-AI is a supervised model with target labels that were derived from the manual screening data of over 50,000 patients who self-reported on the validated Drug Abuse Screening Test [23] and answered follow-up questions about opioid use. SMART-AI is publicly available to run the trained model [24], and more details about the model architecture and development can be found in the original development and validation publication [8]. The model’s development and validation followed guideline recommendations [25].

Temporal validation of the classifier (trained on data between 2017 and 2019 and tested on data from 2020) at an outside hospital demonstrated an area under the precision-recall curve of 0.87 (95% CI 0.84-0.91) for screening for opioid misuse. Similar results were derived in an external validation at a second independent health system [8]. Multiple cutoff points were examined for the optimal threshold selection for the BPA, including the point on the area under the receiver operating curve that minimized the difference between sensitivity and specificity. During validation on the full cohort of hospitalized patients, the optimal cutoff point for screening for opioid misuse was 0.05. At that cutoff, the sensitivity was 0.87 (95% CI 0.84-0.90), specificity was 0.99 (95% CI 0·99-0·99), negative predictive value was 0.99 (95% CI 0.99-0.99), and positive predictive value was 0.76 (95% CI 0.72-0.88). The number needed to evaluate was 1.4, which translates to 26 alerts per 1000 hospitalized patients [8]. This was deemed an acceptable workload for consultation requests in live production for the UW Addiction Medicine clinicians. Additional silent testing was performed at the UW Health to examine sensitivity and specificity with 95% CI in our practice setting.

All notes from the first 24 hours of arrival at the UW Hospital were combined into a single document per patient encounter and converted into sequences of vector representations (eg, embeddings). The CUI embeddings defined the input layer to the SMART-AI model at the encounter level. The model provided prediction probabilities for opioid misuse and stored them in a Databricks table with the predefined cutoff point for screen positives.

#### Component 5: Real-time Delivery of the Prediction Results

The Nebula Cloud Platform was Epic’s Software as a service platform for integrating new technology and specifically supported clinical prediction modeling. Nebula capabilities included the deployment of machine learning models, including a library of Epic-curated models for health care and custom algorithms. Our solution leveraged the latter to facilitate triggers from Epic to call out to the Databricks environment and provided the predictions for BPAs.

**Figure 2:**
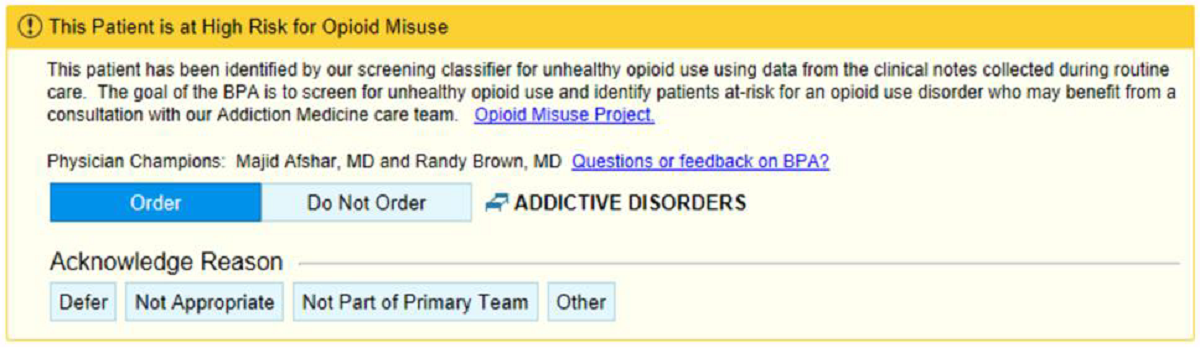
In an iterative design with feedback from end users, a final BPA was implemented for bedside care. The BPA triggers upon opening a chart for a patient that meets the cutpoint predicted probability for opioid misuse from the NLP and deep learning model (SMART-AI). BPA: best practice alert; NLP: natural language processing; SMART-AI: Substance Misuse Algorithm for Referral to Treatment Using Artificial Intelligence.

In the case of SMART-AI, we designed a BPA (Figure 2) to trigger once a clinician opened a patient chart in the EHR. Epic called its Nebula component to determine whether a BPA should be generated. Nebula made an HTTP call to Databricks to request the score. The RESTful HTTP API provided the SMART-AI model score that was serviced using MLFlow. The parameters included UMLS dictionaries, model results, patient identifiers, and other attributes necessary for individual-level predictions. The score was returned to Nebula, which was used to trigger a BPA if SMART-AI met the cutoff score for opioid misuse. For screen positives, the alert recommended the clinician to consult with the UW’s Addiction Medicine consult service. The following were internal targets to meet the real-time needs of the end user at the bedside: (1) a throughput of 1000 notes per minute (<60 ms each); (2) three-nines (99.9%) availability—equivalent of <9 hours of downtime annually; and (3) an established error rate threshold.

#### Component 6: Cybersecurity

Two principles of security were applied: (1) defense in depth and (2) zero trust. The zero-trust architecture was outlined in the National Institute of Standards and Technology Special Publication 800 to 207 [26]. To secure access between Azure Databricks MLFlow and Epic’s Nebula, we used an authentication token and IP range restriction (Databricks admin utility). The authentication token was issued via Databricks standard authentication. As a security best practice, we used the Databricks service principal and its Databricks access token to provide automated tool and system access to Databricks resources.

### Implementation Framework

The Consolidated Framework for Implementation Research informed the development of the preimplementation assessments and will be used during the rapid Plan-Do-Study-Act (PDSA) cycles after deployment [27]. Key stakeholder interviews were planned to better understand the context and identify the barriers to and facilitators of the implementation of the BPA tool. Selected implementation strategies from the Expert Recommendations for Implementing Change were chosen to overcome barriers [28]. For pilot implementation, a regular cadence of meetings was planned with the implementation team to process, reflect, and evaluate the barriers to the implementation and use of the BPA. Process evaluation would incorporate interviews with providers and addiction specialists to understand what barriers still existed to using and acting on the BPA. During pilot implementation, we will collect and summarize clinical performance data during PDSA cycles to guide clinicians and administrators in monitoring, evaluating, and modifying provider behavior. Using the Consolidated Framework for Implementation Research–Expert Recommendations for Implementing Change matching tool [29], we will tailor relevant implementation strategies to enhance provider uptake and use of the tool. In addition, during the pilot phase, we will interview providers on the hospital units beyond the pilot units to identify and explore their determinants for the use of the BPA. After a pilot implementation period of 3 months, we will optimize provider training, enhance educational materials, and institute quality monitoring preparatory to a hospital-wide rollout.

### Patient Outcome Analysis and Power Calculation

The SMART-AI study intervention sample consisted of all hospitalized patients who were screened positive for opioid misuse through the NLP CDS tool. The primary effectiveness measure was the percentage of hospitalized patients in the NLP CDS intervention sample who screened positive for opioid misuse and received an intervention by the inpatient addiction consult service. A control sample was derived by retrospectively applying the NLP CDS tool to all inpatient EHR records from the 2 years before this study’s initiation in March 2023. Hospitalized patients who were screened positive retrospectively through the NLP CDS tool will form the usual care control group.

The primary outcome was the percentage of inpatients who were screened positive (or would have screened positive) through the NLP CDS tool and who received an addiction consult with any of the following interventions: (1) receipt of opioid use intervention or motivational interviewing (MI), (2) receipt of medication-assisted treatment (MAT), or (3) referral to substance use disorder treatment. The primary outcome will be reported as a percentage in the preintervention and postintervention periods and will be measured through substance use screening and treatment service engagement for hospitalized patients screened for opioid misuse. The secondary outcomes included the 30-day unplanned hospital readmission rate. The criteria for unplanned hospital readmissions were adopted from the Centers for Medicare and Medicaid Services [30].

Hypothesis testing for intervention effects will be conducted using independent tests of the difference in the proportion of patients receiving MI, MAT, or referral to substance use disorder treatment. The null hypothesis was that the proportion of patients who screened positive and received any of the aforementioned interventions was lower (inferior) in the postintervention period than in the preintervention period, that is, H_0_: p_1_ − p_2_ ≥ M, where M denotes the noninferiority (eg, equivalence) margin, p_1_ denotes the preperiod proportion_,_ and p_2_ denotes the postperiod proportion. The alternative 1-tailed test for noninferiority, that is, H_1_: p_1_ − p_2_ < M, will be tested using the *Z* statistic. The noninferiority design was adopted to demonstrate that comprehensive screening may be as effective as manual screening but less costly via automated solutions. Our use case was an example of an AI system intended to improve efficiency and throughput within a reasonable timeframe for hospital operations. In these cases, statistically superior performance on outcomes may not be expected or required for prospective implementation, and interventions may be desirable if they are both substantially equivalent (noninferior) on clinical outcomes and cost-effective, given the high cost of building IT infrastructure, hiring vendors, and obtaining licensing and software support.

In hospital-wide screening, we expected a prevalence of 3% of adult inpatients with opioid misuse based on prior findings of hospital-wide analyses. A total sample size of 12,500 patients, with 10,000 in the preintervention 2-year period and 2500 in the postintervention 6-month period, had 85% power to detect a difference of +0.75% in the postintervention period (3.75%) compared with the preintervention period (3%), with a noninferiority difference of −0.5% using a 1-sided *Z* test with a significance level of 0.025.

### Cost-effectiveness Analysis

#### Overview

Cost-effectiveness analysis will estimate the incremental costs of the SMART-AI intervention for the 6 months after the implementation compared with the 6 months before the implementation (ie, the added costs of the SMART-AI tool in reference to usual care) relative to the incremental effectiveness for the primary and secondary outcomes. The health economic evaluation would determine incremental intervention costs by examining the following: (1) the opportunity start-up costs of implementing the SMART-AI tool, (2) the incremental medical costs resulting from usual care for hospitalized patients with opioid misuse versus SMART-AI automated screening–supported care costs, and (3) the ongoing costs of administering and maintaining the SMART-AI tool.

The start-up costs of establishing SMART-AI substance use screening care would include the costs associated with developing and implementing the NLP CDS tool: (1) the cost of supporting the NLP and machine learning components and building the BPA in the EHR and (2) the cost of training the health professionals on tool use. The incremental costs between usual care and SMART-AI automated screening care were determined by calculating medical care costs before and after the implementation of SMART-AI. Medical costs associated with the hospitalization stay and all subsequent medical costs for the 30 days following hospital admission for the pre– and post–SMART-AI intervention periods were derived from hospital billing records and presented from the single-payer (a health system) perspective.

The following 3-pronged approach will be applied to identify the administration and maintenance costs associated with SMART-AI screening workflow changes introduced by the NLP CDS tool: (1) conducting in-depth interviews with hospital administrators, (2) performing activity-based observations of health care personnel who use SMART-AI, and (3) querying the clinician messaging system in the EHR. Average hospital compensation rates were used for valuing health care personnel time costs. Research-related costs were excluded.

#### Analytical Approach to Cost-effectiveness Analysis

The cost-effectiveness analysis was reported in terms of the incremental cost-effectiveness ratio (ICER) per additional patient who received substance use treatment. For this study, the ICER was calculated as the difference between preimplementation and postimplementation intervention costs divided by the difference between preimplementation and postimplementation intervention effectiveness as measured by the rates of patient engagement with substance use treatment services (ie, primary outcome) and 30-day hospital readmission (ie, secondary outcome).

**Figure 3:**
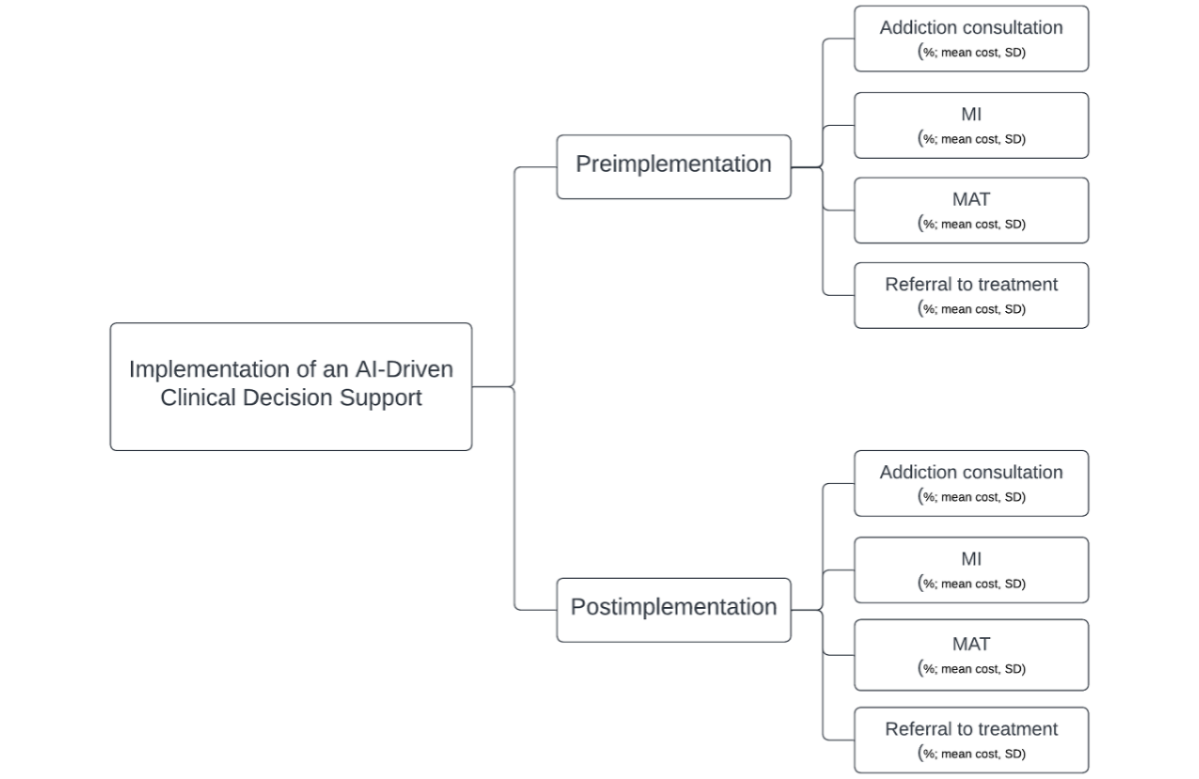
Cost-effectiveness decision tree. AI: artificial intelligence; MAT: medication-assisted treatment; MI: motivational interviewing.

The usual care control group and SMART-AI intervention group were characterized by the pathway probabilities of receiving substance use treatment and meeting the primary outcome. The pathway probabilities of patients’ engagement with inpatient substance use consult, brief intervention or MI, MAT, and referral to substance use treatment for both study groups would result in 8 treatment combinations, which are displayed in Figure 3.

The differential costs pre– and post–SMART-AI intervention were determined as the difference in the weighted sum of the individual pathway costs, using the pathway probabilities as weights for the intervention and control groups. Effectiveness was determined as the difference in the rates of hospitalized patients engaging with substance use disorder treatment before and after the implementation of SMART-AI for the intervention and control groups. The ICER was calculated as follows:



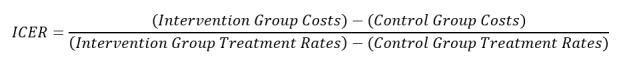



Sensitivity analyses will introduce uncertainty in substance use treatment receipt rates and costs for the intervention and control groups. The Monte Carlo–based simulation estimation used the rates of substance use treatment service uptake observed in the intervention and control groups as a reference to simulate a cohort of postimplementation hospitalized patients and a cohort of usual care hospitalized patients. The ICER per additional individual who received an inpatient substance use consult, brief intervention, MI, MAT, or referral to substance use treatment was calculated by drawing a random sample with replacement from the observed distributions for health care costs (µ_COSTi_) and substance use treatment services (µ_TRTi_) for the intervention and control groups. This process was repeated (n=1000) to produce bootstrap estimates of the 95% CI for the ICER per additional individual who received an inpatient substance use consult, brief intervention, MI, MAT, or referral to substance use treatment. These probabilistic sensitivity analyses estimated the elasticity of the differential cost per patient relative to the differential substance use treatment service rates for the intervention and control groups.

### Ethics Approval

This clinical study was reviewed by the UW Institutional Review Board (ID 2022-0384). The study was part of a larger quality improvement initiative at the UW Health and met the exemption status for human participant research according to the UW Institutional Review Board. The study was secondary research with the collection of existing EHR data that met category 4 exemption. The study met the requirements for a waiver of consent, and all study results were anonymized or deidentified. No compensation was provided in the human participant research.

## Results

### Preimplementation Testing and Approvals

Early-stage investigations were performed to assess the AI system’s predictive performance in a retrospective setting and evaluate the human factors surrounding the BPA before initiating the quasi-experimental clinical study. During the silent testing of SMART-AI at the UW Health, a random sample of 100 adult patient encounters (with an oversampling of patients with the International Classification of Diseases codes for substance use) in 2021 were extracted and reviewed by an inpatient physician and a clinical informatics expert. SMART-AI performed similarly to previously published reports for screening for opioid misuse, with a sensitivity of 93% (95% CI 66%-99%) and specificity of 92% (95% CI 84%-96%).

**Figure 4:**
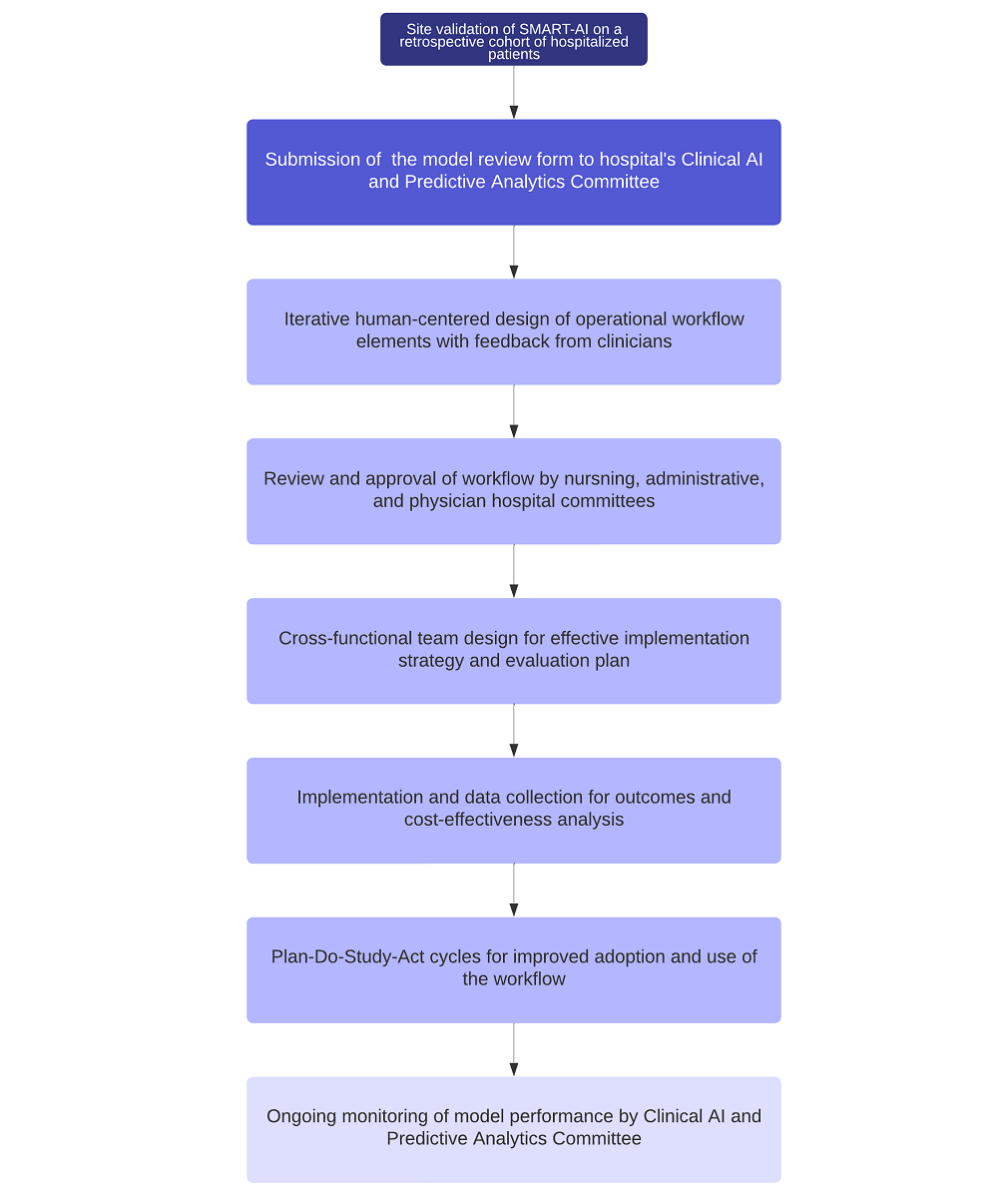
Flow diagram for the process to bedside implementation and evaluation. AI: artificial intelligence; SMART-AI: Substance Misuse Algorithm for Referral to Treatment Using Artificial Intelligence.

Before the deployment of SMART-AI, approvals were received across hospital committees for inpatient operations, EHR super users, CDS, and nursing documentation. The proposal protocol was also reviewed by the Center for Clinical Knowledge Management to confirm that there were no competing interests or roles with existing protocols for screening for substance use conditions in the health system. In addition, SMART-AI was reviewed by the UW’s Clinical AI and Predictive Analytics Committee. A model review form providing details on the clinical problem, model value proposition, model description, proposed workflow integration, internal validation, and monitoring strategy (including fairness and equity) was reviewed and approved by a multidisciplinary committee of clinicians, informaticians, bioethicists, executive leadership, and data scientists. The planned workflow from introduction to implementation is shown in Figure 4.

### Implementation Framework

An end-user interview guide and survey were developed to examine the user acceptability of the BPA. Open-ended questions were asked about the barriers to and facilitators of the use of the BPA. A total of 5 interviews were conducted (with 3 nurse practitioners, 1 family medicine resident, and 1 surgical attendant), and the responses led the production team to create an educational flyer, modify the BPA with more details and options for consultation refusal, and modify when and where the BPA would trigger. Figure 2 shows the final production version of the BPA for deployment. Dissemination efforts included Grand Round presentations to the Addiction Medicine Division, Department of Family Medicine, and notification via the hospital’s weekly electronic newsletter.

The longest delay in operational workflow and architecture was for receiving cybersecurity approvals, especially for the exchange of protected health information between the Microsoft and Epic cloud vendors. An additional 6 months of delay occurred for achieving acceptable security monitors and checks. The go-live of SMART-AI in the EHR was scheduled for January 2023.

## Discussion

### Principal Findings

We offer one of the first protocols that detailed the components of a real-time NLP-driven CDS system for health care delivery at the bedside. We further detailed an implementation framework with human-centered design principles and a planned iterative process to evaluate the cost-effectiveness and health outcomes of screening for opioid misuse. We shared the components and pseudocode with open-source technologies involved in the implementation of an end-to-end NLP pipeline that processed the notes entered by the provider and returned a BPA within minutes for patients at risk of an opioid use disorder. Interviews and user-centered design as well as educational efforts for improving adherence led to changes in the BPA. Finally, we shared an experimental design with a rapid PDSA cycle and cost-effectiveness setup with a noninferiority design to evaluate the screening system for continued implementation or deimplementation.

The digital era in medicine continues to grow exponentially in terms of both the quantity of unstructured data collected in the EHR and the number of prediction models developed for detection and diagnostic, prognostic, and therapeutic guidance. In parallel, the clinical NLP field has grown in its capabilities with the advent of transformer architectures and more affordable and efficient cognitive computing of big data [31]. However, a major bottleneck remains in the successful implementation of NLP and deep learning models in clinical practice. Much of the progress in NLP has focused on information retrieval and extraction [32]; however, the application of these methods at scale with a combination of software developers and operations remains challenging at health care institutions. The role of NLP in BPAs has been limited to date, and prior BPAs have used existing technologies embedded into the EHR [33]. Similar to prior motivations for BPAs delivered to bedside clinicians [34], our intention was to support and enhance decision-making at the beside with a recommendation for an Addiction Medicine consult in patients who may otherwise not receive it or have it delayed, similar to another NLP-driven BPA [17]. However, given the lack of capacity of many EHR vendors to incorporate custom NLP models, we offer an interoperable pipeline to integrate external AI tools with existing EHRs.

Applied clinical NLP has predominantly remained a rule-based approach, but statistical machine learning models are now the leading method in the research literature [21]. Few vendors who provide NLP services rely entirely on machine learning, and a gap remains in effectively applying NLP models to EHRs that go beyond disease detection, which is limited to explicit keyword mentions [35]. Several barriers exist with neural language models, including the need to remove protected health information so that the trained models may be shared and the computational requirements to run complex deep learning models in a production environment [9]. We offer solutions for both barriers using a feature engineering approach to map free text to coded vocabulary and describe a large computing infrastructure with a connection between a data science cloud platform and the EHR to support direct data feeds into any machine learning model. The NLP CDS pipeline accomplishes efficiency in data standardization and scalability [36] for successful implementation and is extensible to other NLP engines. The benefit of augmented intelligence remains unknown and its identification using our health care outcomes and cost-effectiveness analysis is the next step in a clinical study.

Our implementation framework is largely guided by a team of implementation scientists supported by the UW’s Clinical and Translational Science Award. We leveraged our Clinical and Translational Science Award’s Dissemination and Implementation Launchpad to help bridge the gap between evidence-based research and practice [37]. The Dissemination and Implementation Launchpad serves to accelerate the pace of disseminating research findings and increase the adoption and implementation of effective interventions, leading to sustainable practice and policy changes. It uses strategies from implementation science, design thinking, and human-centered engineering for the better integration of AI technologies into health systems. As part of the preimplementation phase, we assessed contextual factors that may impact implementation by engaging both adopters, who are the decision-makers, and end users, who are the main implementers, of the tool [38]. We conducted qualitative interviews with end users to evaluate the need for the tool and BPA design. We involved adopters early in the process to inform the intervention or implementation process through consultations during the design, feasibility testing, and implementation phases. An iterative process ensued to address the constraints and contextual factors that affect the adoption and implementation of the tool in our health system.

During the preimplementation phase, the project team clarified roles with the project management, with the readiness of the clinical workflow approved through hospital committee meetings and individual interviews with end users. Our health system is an early adopter of AI governance with a review process similar to that of other health systems [39]. The Clinical AI and Predictive Analytics Committee follows the Minimum Information About Clinical AI Modeling checklist [40]. The offline validation of our model incorporated principles from multiple reporting guidelines on prediction models, bias, fairness, and validation [41]. Clinical evaluation after the go-live of SMART-AI will follow the reporting guideline for the early-stage clinical evaluation of decision support systems driven by AI (Developmental and Exploratory Clinical Investigations of Decision Support Systems Driven by AI) [42].

The build of an enterprise-wide AI infrastructure for data-driven CDS is an important feature of a data-driven learning health system. At the UW, learning health system activities dating back to 2013 established an evidence-based framework with a series of organizational-level quality improvement interventions [43]. In 2020, the UW Health reaffirmed its strategic plan for embedding discovery and innovation as well as diversity, equity, and inclusion in clinical care. Successful implementation included coaching staff and administrative leaders for working in PDSA with lean management to get the problem, analysis, corrective actions, and action plan down on a single sheet of large (A3) paper, also known as “A3” thinking [44]. A rapid PDSA cycle is important in the advent of AI-driven interventions that require rigorous evaluation for implementation or deimplementation. Furthermore, the pipeline developed for the opioid screener use case is applicable to other CDS tools that use machine learning and NLP. We designed our architecture to ingest different modalities of data and provide a computing environment that is flexible to different data modalities and machine learning algorithms.

Several limitations exist in the deployment and sustainability of our NLP-driven CDS tool. First, calibration drift is a real concern with changes in medical practice, evidence, and demographic shifts over time that may affect model performance [45]. During implementation, reviews by the Clinical AI and Predictive Analytics Committee will include quarterly evaluations of the sustained effectiveness of the tool, an audit of the fairness of the tool across parity groups, and examination for alert fatigue. Others have shown benefits in recalibration approaches and domain adaptation with additional training data to update the models over time [46]. Furthermore, the start-up costs of the pipeline may be cost-prohibitive for small health systems. Our proposed cost-effectiveness analysis will provide a perspective on both the start-up costs of implementing the NLP tool and the ongoing incremental costs. The start-up costs are more of a burden to a small health system than the incremental costs, but we expect that our results will be informative in terms of both these costs.

### Conclusions

The deployment of medical AI systems in routine clinical care presents an important yet unfulfilled opportunity [47], and our protocol aims to close the gap in the implementation of AI-driven CDS. Our protocol implementation for an enterprise-wide production environment of an AI opioid misuse screener provides a model for other health systems to use to bring NLP models into practice for CDS. We highlight opportunities to leverage the expertise of our applied data science team to use the open-source tools for feature engineering and model development inside a larger infrastructure with vendor support for hardware and software dependencies. Given the sensitive nature of health care data, the biggest challenges are ensuring high standards for cybersecurity and meeting the privacy requirements for protecting patient data.

## Appendix

Multimedia Appendix 1 Pseudocode for custom Python scripts.

## Abbreviations

AI: artificial intelligence
API: application programming interface
BPA: best practice alert
CDS: clinical decision support
CNN: convolutional neural network
cTAKES: Clinical Text Analysis and Knowledge Extraction System
CUI: concept unique identifier
EHR: electronic health record
HL7: Health Level 7
ICER: incremental cost-effectiveness ratio
MAT: medication-assisted treatment
MI: motivational interviewing
MLFlow: machine learning model life cycle management
NLP: natural language processing
PDSA: Plan-Do-Study-Act
SMART-AI: Substance Misuse Algorithm for Referral to Treatment Using Artificial Intelligence
UMLS: Unified Medical Language System
UW: University of Wisconsin

## Data Availability

The raw electronic health record data are not available because of regulatory and legal restrictions imposed by the University of Wisconsin-Madison Institutional Review Board. The original data were derived from the institution’s electronic health record and contain patients’ protected health information. Data are available from the University of Wisconsin Health Systems for researchers who meet the criteria for access to confidential data and have a data use agreement with the health system. Only the final trained model that is fully deidentified with a vocabulary of mapped concept unique identifiers is open source and available [38]. Our deidentification approach has been previously described [39].
