# Supplementary material for: Deployment of Real-time Natural Language Processing and Deep Learning Clinical Decision Support in the Electronic Health Record: Pipeline Implementation for an Opioid Misuse Screener in Hospitalized Adults
Source: JMIR Med Inform. 2023 Apr 20;11:e44977. doi: 10.2196/44977 (PMC10160938; doi:10.2196/44977)
Supplement: Multimedia Appendix 1 [file medinform_v11i1e44977_app1.docx]

**Appendix. Pseudocode for Independent Services to ingest, score, and report the NLP model** The code does not include the processing of the notes nor the trained model. Those are open-source and the online resources are reported separately in the manuscript

**HL7Listener**

The purpose of the HL7 Listener is to respond to data pushed to it by Cloverleaf, parse the message and store relevant parts both to an encounter database table and to a file containing only the note text

When a new clinical note is authored by a clinician, Cloverleaf sends it to the HL7Listern service using TCP/IP:

WHEN ***BYTESTREAM*** received over TCP/IP:

# BYTESTREAM is in HL7v2.3 pipe-delimited format; parse out the parts we need

*contactSerialNumber, noteID, noteText, inpatientStatus, patientDOB, admissionServiceLocation, inpatientStatus, patientAge, admissionServiceLocation* = parse(*BYTESTREAM*)

*patientAge =* CURRENT_DATE() *- patientDOB*

IF *timeSinceAdmission* < 24 hours AND *patientAge* >= 18

AND *admissionServiceLocation* = “University Hospital”:

INSERT *contactSerialNumber, noteID, noteText* INTO TABLE ***cnlp.EncounterText***

WRITE *noteText* TO FILE ***contactSerialNumber*** IN DIRECTORY ***CTAKES_INPUT***

**Scoring**

The purpose of the Scoring module is to run CTAKES on files containing clinical notes to create CUIs, and then run SMART-AI on those CUIs to generate encounter scores. The results are stored in a database table

EVERY 5 MINUTES:

CALL_USING_SHELL **CTAKES**

with INPUT DIRECTORY ***CTAKES_INPUT*** and OUTPUT DIRECTORY ***CTAKES_OUTPUT***

*newcuis* = pandas.DataFrame()

FOR EACH FILE *file* in ***CTAKES_OUTPUT***:

*cui_string = PARSE_AND_EXTRACT_CUIS(file)*

*data =* {'csn': int(*file*.name), 'concepts': *cui_string* }

*newcuis.append(data)*

*p* = SMART_AI.predict_batch(*newcuis*) # Score cuis with SMART-AI

data = {'csn': *p.csn*, *p.opioid* }

INSERT *data* INTO TABLE ***cnlp.EncounterScores***

**MLFlowRESTAPIService**

This service is a RESTful service that reports scores for a given encounter:

WHEN ***contactSerialNumber*** received over HTTP:

SELECT *csn*, *scores* FROM ***cnlp.EncounterScores*** WHERE csn = contactSerialNumber

RETURN as httpPost(*csn, scores*)
